# Small RNA GadY in *Escherichia coli* enhances conjugation system of IncP-1 by targeting SdiA

**DOI:** 10.3389/fcimb.2024.1445850

**Published:** 2024-07-23

**Authors:** Shebin Zhang, Jiao Long, Qiwei Li, Mo Li, Ruiqi Yu, Yang Lu, Xingyan Ma, Yimei Cai, Cong Shen, Jianming Zeng, Bin Huang, Cha Chen, Jieying Pu

**Affiliations:** ^1^ The Second Clinical Medical College, Guangzhou University of Chinese Medicine, State Key Laboratory of Traditional Chinese Medicine Syndrome, Guangdong Provincial Hospital of Chinese Medicine, Guangzhou, China; ^2^ Department of Laboratory Medicine, The Second Affiliated Hospital of Guangzhou University of Chinese Medicine, Guangzhou, China; ^3^ Department of Laboratory Medicine, The First Affiliated Hospital of Sun Yat-sen University, Guangzhou, China; ^4^ Guangdong Provincial Key Laboratory of Research on Emergency in TCM, Guangzhou, China

**Keywords:** GadY, small RNA, plasmid conjugation, *Escherichia coli*, SdiA

## Abstract

Plasmid-mediated conjugation is a common mechanism for most bacteria to transfer antibiotic resistance genes (ARGs). The conjugative transfer of ARGs is emerging as a major threat to human beings. Although several transfer-related factors are known to regulate this process, small RNAs (sRNAs)-based regulatory roles remain to be clarified. Here, the Hfq-binding sRNA GadY in donor strain *Escherichia coli* (*E. coli*) SM10λπ was identified as a new regulator for bacterial conjugation. Two conjugation models established in our previous studies were used, which SM10λπ carrying a chromosomally integrated IncP-1α plasmid RP4 and a mobilizable plasmid pUCP24T served as donor cells, and *P. aeruginosa* PAO1 or *E. coli* EC600 as the recipients. GadY was found to promote SM10λπ-PAO1 conjugation by base-pairing with its target mRNA SdiA, an orphan LuxR-type receptor that responds to exogenous N-acylated homoserine lactones (AHLs). However, SM10λπ-EC600 conjugation was not affected due to EC600 lacking AHLs synthase. It indicates that the effects of GadY on conjugation depended on AHLs-SdiA signalling. Further study found GadY bound SdiA to negatively regulate the global RP4 repressors KorA and KorB. When under ciprofloxacin or levofloxacin treatment, GadY expression in donor strain was enhanced, and it positively regulated quinolone-induced SM10λπ-PAO1 conjugation. Thus, our study provides a novel role for sRNA GadY in regulating plasmid-mediated conjugation, which helps us better understand bacterial conjugation to counter antibiotic resistance.

## Introduction

Conjugation is a common mechanism for horizontal gene transfer in bacteria. It is a process by which plasmid DNA is transferred from one cell (donor) to another cell (recipient) by direct contact (Llosa et al., 2002). The dissemination of antibiotic resistance genes (ARGs) by bacterial conjugation is one of the main causes of antibiotic resistance (Cabezón et al., 2017). Most ARGs are encoded on mobile genetic elements and have been found on conjugative plasmids (McInnes et al., 2020). The plasmids carry one or more ARGs for all major classes of antibiotics currently used in clinical treatments, and they can also serve as a reservoir of ARGs to maintain antibiotic resistance even in the absence of antibiotic selection (von Wintersdorff et al., 2016; Vrancianu et al., 2020). Thus, conjugation-mediated antibiotic resistance is an accelerating crisis in bacteria, and understanding the mechanisms of bacterial conjugation is crucial for developing strategies to cope with the crisis.

The entire process of conjugation is tightly controlled by several transfer-related proteins, including TraJ, the major transcriptional activator of the transfer (tra) operon, and relaxosome components such as TraI, TraH and TraM (Pansegrau et al., 1990, 1993; Koraimann et al., 1996). Besides, the KorA and KorB proteins are present as global repressors leading to an inhibition in bacterial conjugation (Kostelidou et al., 1999). KorA inhibits seven operons including trfA on the IncP-1 plasmid RK2 (Jagura-Burdzy and Thomas, 1994). KorB acts cooperatively with KorA to function as a regulator protein in the expression of transfer genes by down-regulating the transcription of the *trfA*, *kilA* and *korAB* operons (Balzer et al., 1992). The mRNA expressions of these proteins are regulated at both transcriptional and post-transcriptional levels (Pansegrau et al., 1994).

Small RNAs (sRNAs) have emerged as important transcriptional and post-transcriptional regulators in bacteria in the past years (Parmeciano Di Noto et al., 2019). Bacterial sRNAs are usually between 40 and 500 nucleotides (nt) in length and most are transcribed from intergenic regions (Li et al., 2012). Under specific conditions, sRNAs expressions are generally modulated and help the bacteria rapidly adapt to environmental changes. Similar to microRNAs, their functional analogs in eukaryotes, sRNAs regulate several aspects of gene expression including transcription, translation, and mRNA stability by base-pairing with multiple target mRNAs (Dutta and Srivastava, 2018). These functions of sRNAs usually require the help of an RNA chaperon, Hfq (Murina and Nikulin, 2015; Santiago-Frangos and Woodson, 2018). Hfq is a potent regulator of many aspects of bacterial activities by influencing mRNA translation and stability via binding with sRNAs (Vogel and Luisi, 2011), and it has been reported to affect the transfer of conjugative plasmids by modulating traJ and traM transcript stability (Will and Frost, 2006). Given the close relationship between sRNAs and Hfq, the Hfq-binding sRNAs have attracted considerable attention recently. Quite a few Hfq-binding sRNAs in *E. coli* were identified (Gottesman, 2004; Faner and Feig, 2013; Kavita et al., 2018). Many of these sRNAs still have uncharacterized functions. In particular, whether the role of Hfq in bacterial conjugation has anything to do with these binding sRNAs is still unclear. The roles of Hfq-binding sRNAs in conjugation need further investigation.

GadY is an Hfq-binding sRNA in *E. coli*, and has been reported in the past as a potent regulator of acid response (Opdyke et al., 2004). The gene encoding this sRNA, *gadY*, is located between the *gadX* and *gadW* coding sequences on the opposite strand of the chromosome (Tramonti et al., 2008). When bacteria face mild acidic conditions, GadY is activated and binds its target mRNA GadX in a base-pairing pattern to regulate the glutamate decarboxylase encoded genes *gadA* and *gadB*, and the glutamate/GABA antiporter encoded gene *gadC* (Opdyke et al., 2011; Le Vo et al., 2012). It was also reported that GadY reduces acetate production and improves bacterial growth when *E. coli* under a mild acidic condition (Negrete and Shiloach, 2015). These current studies identify GadY’s roles in acid resistance, other biological functions of GadY however remain to be elucidated. Our previous works found the orphan LuxR-type receptor SdiA in *E. coli* SM10λπ regulated bacterial conjugation by responding to exogenous N-acylated homoserine lactones (AHLs) produced by *P. aeruginosa* PAO1 (Lu et al., 2017b), and the expression of GadY was affected by SdiA when *E. coli* was under acid stress (Ma et al., 2020). Whether GadY is involved in the regulation of bacterial conjugation remains to be elucidated.

To clarify the role of GadY in bacterial conjugation, gadY gene-deficient and overexpressing donor strains were generated in *E. coli* SM10λπ based on the conjugation models established in our previous studies (Lu et al., 2017b; Xiong et al., 2021), and PAO1 or *E. coli* EC600 were used as recipient strains. The *E. coli* SM10λπ carries a chromosomally integrated IncP-1α plasmid RP4. There is also a pUCP24T plasmid in SM10λπ, and it is mobilizable with the help of RP4 providing transfer functions. In this study, we demonstrated that GadY in donor strain promoted SM10λπ-PAO1 conjugation by base-pairing with its target mRNA SdiA. Our study provides a novel role for the Hfq-binding sRNA GadY in regulating bacterial conjugation, with implications for fighting antibiotic resistance by developing anticonjugation compounds.

## Materials and methods

### Bacterial growth conditions

The bacterial strains and plasmids used in this study are described in **Supplementary Table S1**. *E. coli* K12 SM10λπ (LMBP 3889) is available at BCCM/GeneCorner. To culture the bacteria, strains were grown in Luria-Bertani (LB) medium with shaking or on LB plates containing 1.5% agar at 37°C. In the experiments of SM10λπ-PAO1/EC600 conjugation, antibiotics were added with the following concentrations: 30 μg/ml gentamicin (GM), 50 μg/ml rifampin (RIF), 100 μg/ml ampicillin (AMP), and 16 μg/ml chloramphenicol (CM). In the experiment with exogenous AHLs replenishment, 40 µM 3-oxo-C12-HSL and 40 µM C4-HSL were used.

### Construction of GadY-deficient mutant and overexpressing strain

GadY-deficient mutant was generated in the SM10λπ strain by using a bacteriophage lambda Red recombination system, following the steps described by Datsenko and Wanner (Datsenko and Wanner, 2000). The recombinogenic plasmid pKD46 mediates lambda Red homologous recombination function. The plasmid pKD3 was used as a template and GadY-knock-F/R (**Supplementary Table S2**) as primers for PCR amplification. Afterwards, 100 μl cells were transformed with 200 ng PCR products using electroporation. Recombinants were selected on LB agar (CM, 16 μg/ml) and then transformed with pCP20 to eliminate the FRT-flanked cat gene. The mutants were verified by PCR with primers GadY-M1/M2 described in **Supplementary Table S2**. Finally, the pSTV28 plasmid which can stably replicate in *E. coli* was transformed into the mutant to construct the △GadY strain. Meanwhile, pSTV28 is used to construct the GadY overexpressing strain as follows: *gadY* gene sequence from *E. coli* WT genomic DNA was amplified by PCR using the cloning primers In28-*gadY*-F/R listed in **Supplementary Table S2**. The product of PCR was inserted into *Eco*RI/*Hin*dIII sites downstream of P*_lac_* in pSTV28. After direct sequencing (Sangon Biotech), the GadY overexpressing plasmid, and pSTV28 plasmid were transformed into SM10λπ respectively to generate the GadY^+^ strain, and the empty vector strain (EV).

### Bacterial conjugation assays

The conjugation experiments in this study were based on the conjugation models established in our previous studies (Lu et al., 2017b; Xiong et al., 2021). *E. coli* SM10λπ worked as donor strains, and EC600 or PAO1 as recipient strains. GadY-deficient and overexpressing strains were constructed in SM10λπ which carries an IncP-1α plasmid RP4 (chromosomally integrated) and a mobilizable plasmid pUCP24T. The pUCP24T plasmid was generated from pUCP24 which was inserted with an *oriT* fragment (West et al., 1994). It is with GM resistance, and is mobilizable with the help of RP4 providing transfer functions. For mating experiments, the donor cells and recipient cells were respectively grown in LB medium overnight, 200 µl of each culture was added to 3 ml fresh LB medium for a second growth of 2 h, and then the cultures were mixed in fresh LB medium with a ratio of 1:1 (1×10^7^ CFU/ml) at 37°C for 6 h under static conditions, total volume of the medium was 200 µl. Next, the cultures were thoroughly mixed, and then 30 µl of each mixture was spread on an LB agar plate containing GM+AMP (SM10λπ-PAO1 transconjugants) or GM+RIF (SM10λπ-EC600 transconjugants). After incubation at 37°C overnight, the numbers of transconjugant colonies were counted. In the experiment of antibiotic-induced conjugation, the overnight cultured donor cells were diluted with LB containing ciprofloxacin (CIP, 0.015625 μg/ml), levofloxacin (LEV, 0.03125 μg/ml), GM (0.3 μg/ml), azithromycin (AZM, 32 μg/ml) or CM (16 μg/ml) and were incubated at 37°C. After 6 h of incubation, the cells were washed and re-suspended in fresh LB medium without antibiotics, and used as donor strains for conjugation.

### qRT-PCR

The bacterial strains were cultured in LB medium at 37°C for indicated time points. After culture collection, the total RNA of bacteria was extracted by TRIzol (Invitrogen) and a spectrophotometer (NanoDrop 2000) was used to quantify the RNA. Then an RT Reagent Kit (PrimeScript, Takara) was applied to reverse transcription, in which 1 μg RNA was used and the total reaction volume was 20 μl. The quantitative PCR was performed using a SYBR Green qPCR kit (Accurate Biology) and the ViiATM 7Dx system (Applied Biosystems). The relative threshold cycle (2^-ΔΔCt^) method was used to normalize the quantification cycle value to the reference gene *rpoD*. The qRT-PCR primers were listed in **Supplementary Table S2**.

### IntaRNA analysis

The online IntaRNA database (Mann et al., 2017) was used to predict the potential targets of GadY. Use the default parameters as described on the IntaRNA website (http://rna.informatik.uni-freiburg.de/IntaRNA/Input.jsp), of which the number of interactions per RNA pair was 5, and minimal number of basepairs in seed was 7. The query sRNA and target sequences were from the genome of *E. coli* K12 SM10λπ. Results are computed with IntaRNA version 3.3.1 linking Vienna RNA package 2.5.0.

### Fluorescent reporter assays

To verify the interaction between GadY and SdiA, the pUCP30T-*gfp* reporter plasmid we previously generated was used (Lu et al., 2019; Pu et al., 2022). It is a plasmid containing a gentamicin resistance gene (aacC1), the origin of replication from pRO1600, oriT, and the GFP reporter region with a *lac* promoter that allows the insertion sequence to express transcriptional fusion with GFP. For WT SdiA plasmid, pUCP30T-*sdiA*-*gfp*, a WT *sdiA* mRNA sequence containing GadY putative binding site from SM10λπ genomic DNA was amplified. Next, the *Xba*I/*Nco*I sites upstream of GFP in the pUCP30T-*gfp* plasmid were inserted with the amplified sequence, and the plasmid was constructed. For Mut SdiA plasmid pUCP30T-mut-*sdiA*-*gfp*, or for Mut GadY overexpressing plasmid pSTV28-mut-GadY^+^, which carried a mutated sequence in the complementary sites for GadY, or SdiA, was generated using fusion PCR. The cloning primers listed were shown in **Supplementary Table S2**. After direct sequencing, the plasmid pSTV28-mut-GadY^+^ was transformed into SM10λπ to generate the Mut GadY^+^ strain, and the plasmid pUCP30T-*sdiA*-*gfp* or pUCP30T-mut-*sdiA*-*gfp* was transformed into SM10λπ EV, △GadY, or GadY^+^ strain respectively, or was transformed into SM10λπ EV, GadY^+^ or Mut GadY^+^ strain respectively. Incubating for 20 h overnight, the bacterial cultures were collected and OD_600_ values were taken, and then were washed and re-suspended in 0.9% NaCl. 100 μl of the solution was then used to determine the fluorescence intensity (F485/535) through a microplate reader (Synergy H1, BioTek), and 10 μl of the solution was applied to observe the fluorescence by a fluorescence microscope (ECLIPSE Ti2-U, Nikon).

### Growth curves

The indicated strains were grown overnight in LB medium at 37°C, and then each 200 μl culture was added to a new 3 ml LB medium for the second growth of 2 h. Next, each culture was 1:1000 diluted and 200 μl was added to an automated 96-well microplate reader (Synergy H1, BioTek) at 37°C growing for 15 h. The OD_600_ values were taken at 1 h intervals with continuous shaking.

### β-Galactosidase assays

A plasmid pQF50 containing the *lacZ* reporter gene which codes for β-galactosidase was used in the study. The DNA sequences of the *korA* promoter (*PkorA*) ranging from -1 to -173 and the *korB* promoter (*PkorB*) ranging from -1 to -328 were amplified by PCR and inserted into the *Hin*dIII site upstream of the *lacZ* gene. The cloning primers were listed in **Supplementary Table S2**. Then the pQF50-*PkorA* and pQF50-*PkorB* plasmids were constructed, and confirmed by direct sequencing (Sangon Biotech). Next, the above two plasmids were respectively transformed into DH5α, an *E. coli* strain without endogenous β-galactosidase, and the pSTV28 (EV) or pSTV28-GadY^+^ (GadY^+^) plasmid was subsequently transferred. In the β-galactosidase assays, the strains were cultured at 37°C for 6 h to the mid-log phase, and the Miller method was carried out to examine the β-galactosidase activity (Giacomini et al., 1992).

### Statistical analysis

At least three independent experiments were performed in each set of individual assays. Data are shown as mean ± SEM by using GraphPad Prism 9. The statistical significance between two groups was determined by the Student’s *t* test, and the *P* value was described as *, *P* < 0.05; **, *P* < 0.01; and ***, *P* < 0.001.

## Results

### Positive regulation of SM10λπ-PAO1 conjugation by GadY

**Figure 1:**
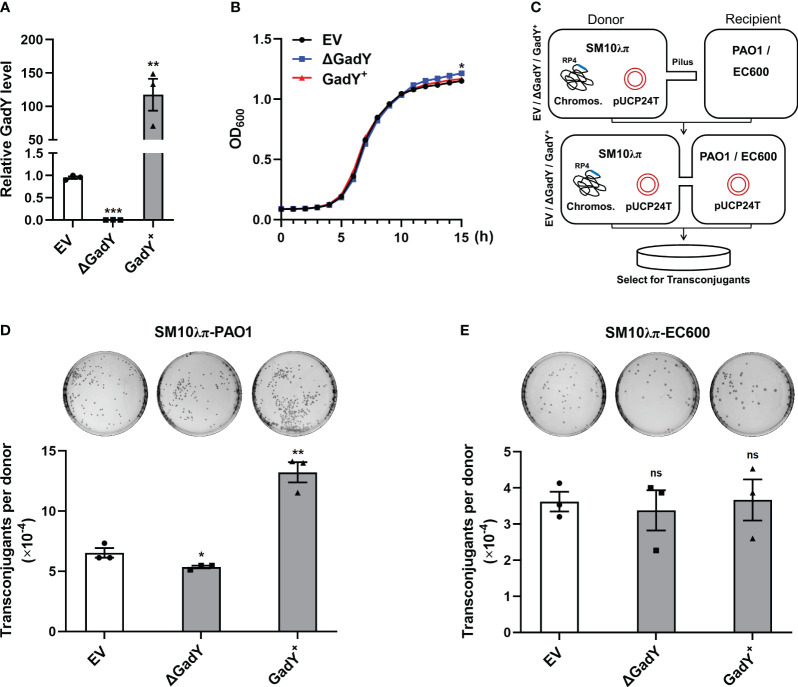
GadY positively regulated SM10λπ-PAO1 conjugation, but it did not affect the conjugation between SM10λπ and EC600. **(A)** *E*. *coli* SM10λπ carrying pSTV28 empty vector or pSTV28-*gadY* plasmid termed EV and GadY^+^ respectively, and *gadY*-deleted SM10λπ carrying pSTV28 plasmid termed △GadY, these three strains were grown in LB for 6 h, then the expression levels of GadY were detected by qRT-PCR. **(B)** Analysis of the growth curves of EV, △GadY, and GadY^+^ strains. **(C)** The conjugation models of SM10λπ-PAO1/EC600. **(D, E)** The donor strains SM10λπ (EV, △GadY, or GadY^+^) and recipient strain PAO1 **(D)** or EC600 **(E)** were mated at 37°C for 6 h (1×10^7^ CFU/ml each), and then the numbers of transconjugant colonies were counted. Data are shown as mean ± SEM of at least three independent experiments. *, *P* < 0.05; **, *P* < 0.01; ***, *P* < 0.001; ns, non-significant.

To explore GadY’s role in bacterial conjugation, the gadY gene-deficient and overexpressing strains were constructed with SM10λπ, termed △GadY and GadY^+^. Fresh LB medium was used to culture the empty vector (EV), △GadY, and GadY^+^ strains for 6 h respectively, and the expression level of GadY was examined by qRT-PCR. GadY expression was up-regulated (~117.5-fold) in the GadY^+^ strain, and was undetected in the △GadY strain (**Figure 1A**). The bacterial growth curves showed little difference between the three strains, of which the △GadY strain grew faster under the stationary phase (**Figure 1B**). SM10λπ-PAO1 and SM10λπ-EC600 were two conjugation models previously generated in our lab (Lu et al., 2017b; Xiong et al., 2021), as shown in **Figure 1C**. We first examined the role of GadY in regulating SM10λπ-PAO1 conjugation. Compared with the EV group, overexpression of GadY significantly enhanced the conjugation between SM10λπ and PAO1, while the GadY deficiency group resulted in a reduction (**Figure 1D**). We subsequently used the SM10λπ-EC600 conjugation model for investigation, but found no statistical significance (**Figure 1E**). These results suggest GadY in SM10λπ donor strains has different roles in regulating conjugation with PAO1 or EC600 recipients.

### Base-pairing between GadY and its target mRNA SdiA

Different roles of GadY in the two conjugation models were found in **Figure 1**. The differences may be caused by the recipient strains PAO1 and EC600. In contrast to *P. aeruginosa*, *E. coli* lacks AHLs synthase and therefore could not produce AHLs (Kumar et al., 2022). While in *P. aeruginosa*, quorum sensing (QS) is a vital AHLs signalling system, in which 3-oxo-C12-HSL and C4-HSL are common signals produced by LasI or RhlI respectively (Lee and Zhang, 2015). The donor SM10λπ is also an *E. coli* strain which does not produce AHLs, but it encodes an orphan LuxR-type receptor SdiA which responds to exogenous AHLs (Yamamoto et al., 2001). Thus, it suggests that the AHLs-SdiA signalling in the SM10λπ-PAO1 model may contribute to the different roles of GadY in the two conjugation models.

**Figure 2:**
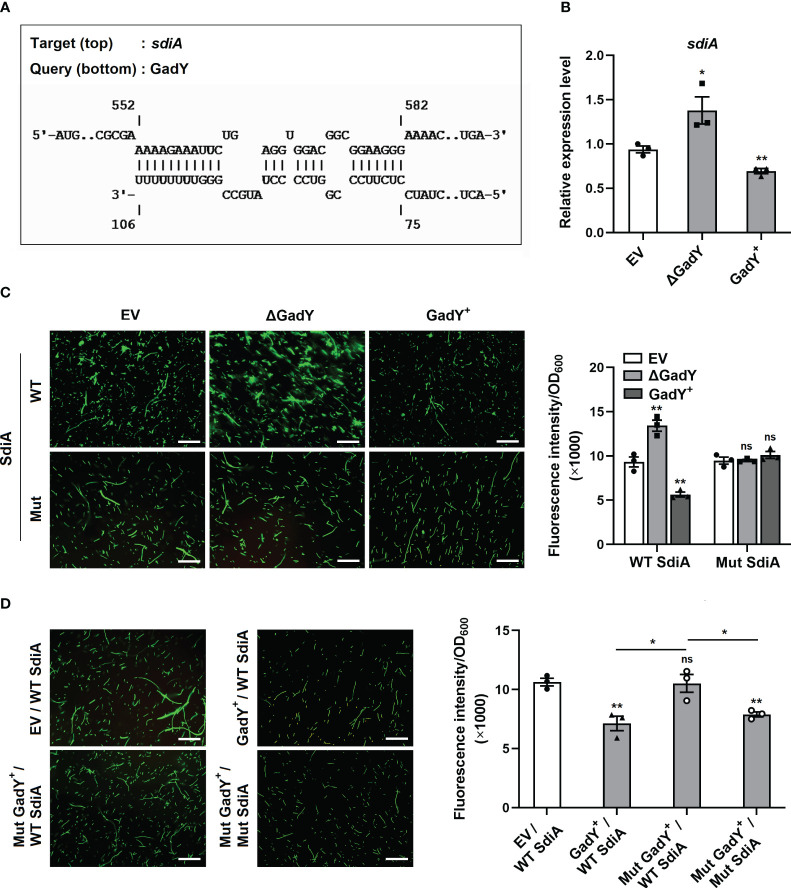
GadY base-paired with its target mRNA SdiA and negatively regulated its transcription. **(A)** Analysis of GadY’s target mRNA through IntaRNA, and found coding sequence of *sdiA* had a putative binding region with GadY. **(B)** *E*. *coli* SM10λπ EV, GadY^+^, and △GadY strains were grown in LB for 6 h respectively, then SdiA mRNA expression was detected by qRT-PCR. **(C, D)** The pUCP30T-*gfp* reporter plasmid was inserted with a WT or Mut sequence of *sdiA* mRNA, and was transformed into SM10λπ EV, △GadY or GadY^+^ strains **(C)**, or EV, GadY^+^ or Mut GadY^+^ strains **(D)**, then the fluorescence images were photographed by a microscopy, the scale bar represents 10 μm, and the fluorescence intensity normalized to OD_600_ was measured by a microplate reader for mapping the bar graphs. Data are shown as mean ± SEM of at least three independent experiments. *, *P* < 0.05; **, *P* < 0.01; ns, non-significant.

Base-pairing with its target mRNAs is an important way for sRNA to regulate various genes. To explore the molecular mechanisms of GadY affecting SM10λπ-PAO1 conjugation, the online IntaRNA database (Mann et al., 2017) was used to predict the potential targets of GadY. Surprisingly, SdiA was found to be a target mRNA, and the base-pairing of GadY occurs at the coding region (**Figure 2A**). The expressions of *sdiA* in EV, △GadY, and GadY^+^ strains were detected by qRT-PCR, and we found a significant up-regulation in the GadY deletion mutant and a decrease in the GadY overexpressing strain (**Figure 2B**). To verify the predicted data of IntaRNA, we developed a GFP (green fluorescent protein) reporter system. The pUCP30T-*sdiA*-*gfp* plasmid was first constructed by inserting the predicted mRNA sequence of *sdiA* upstream of the *gfp* gene. Next, the plasmid was transformed into SM10λπ EV, △GadY, or GadY^+^ strains respectively. A fluorescence microscope was used to observe and photograph the fluorescence, and a microplate reader was applied to detect the fluorescence intensity for mapping the bar graphs. As shown in **Figure 2C**, the intensity of GFP that carried a wild-type (WT) base-pairing site of SdiA was significantly affected by GadY, but the mutant (Mut) groups were not. To further confirm the direct interaction between GadY and SdiA, we also mutated GadY and then introduced a compensatory change in the SdiA base-pairing site to conduct mutational analyses. As expected, overexpressing mutant GadY (Mut GadY^+^) disrupted the inhibition role of GadY in the GFP intensity of WT SdiA, but meanwhile introducing the SdiA compensatory mutant (Mut SdiA) restored GadY’s role (**Figure 2D**). These results indicate that SdiA as a direct target of GadY may be involved in the role of GadY in SM10λπ-PAO1 conjugation.

### Effects of GadY on conjugation depend on AHLs-SdiA signalling

**Figure 3:**
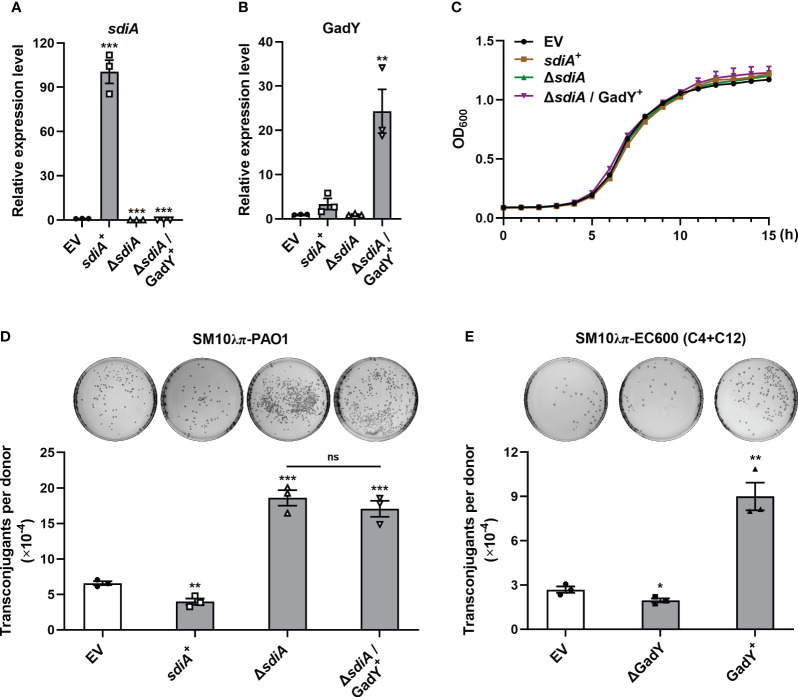
AHLs-SdiA signalling was involved in GadY’s effect on bacterial conjugation. **(A, B)** *E*. *coli* SM10λπ carrying pSTV28 empty vector or pSTV28-*sdiA* plasmid termed EV and *sdiA* ^+^ respectively, and *sdiA*-deleted SM10λπ carrying pSTV28 or pSTV28-*gadY* plasmid termed △*sdiA* and △*sdiA*/GadY^+^ respectively, these four strains were grown in LB for 6 h, then *sdiA* **(A)** and GadY **(B)** expressions were examined by qRT-PCR. **(C)** Analysis of the growth curves of EV, *sdiA* ^+^, △*sdiA*, and △*sdiA*/GadY^+^ strains. **(D)** The donor strains SM10λπ (EV, *sdiA* ^+^, △*sdiA*, or △*sdiA*/GadY^+^) and recipient strain PAO1 were mated at 37°C for 6 h (1×10^7^ CFU/ml each), and then counted the numbers of transconjugant colonies. **(E)** The donor strains SM10λπ (EV, △GadY, or GadY^+^) were cultured in the presence of 40 µM 3-oxo-C12-HSL and C4-HSL, followed by EC600 conjugation, the mixtures were incubated at 37°C for 6 h (1×10^7^ CFU/ml each), and then counted the numbers of transconjugant colonies. Data are shown as mean ± SEM of at least three independent experiments. *, *P* < 0.05; **, *P* < 0.01; ***, *P* < 0.001; ns, non-significant.

The AHLs receptor SdiA was found to be a target of GadY, and its mRNA expression was negatively regulated by GadY in the donor strain SM10λπ. Our previous study has demonstrated the inhibition role of AHLs-SdiA signalling in SM10λπ-PAO1 conjugation (Lu et al., 2017b). To clarify whether SdiA participates in GadY-mediated regulation of conjugation, *sdiA* gene-overexpressing and deficient strains in SM10λπ constructed by our previous work (Lu et al., 2017b) were used, termed *sdiA* ^+^ and △*sdiA*. Overexpression of GadY in *sdiA*-deficient strain was generated, termed △*sdiA*/GadY^+^. After 6 h of culture, the expression levels of *sdiA* (**Figure 3A**) and GadY (**Figure 3B**) in EV, *sdiA* ^+^, △*sdiA*, and △*sdiA*/GadY^+^ strains were confirmed by qRT-PCR. Compared with the EV group, the *sdiA* mRNA expression was up-regulated (~100.5-fold) in *sdiA* ^+^ strain, and was undetected in △*sdiA* or △*sdiA*/GadY^+^ strain (**Figure 3A**). GadY expression was up-regulated (~24.3-fold) in △*sdiA*/GadY^+^ strain compared with the EV group, and its expression in *sdiA* ^+^ or △*sdiA* stains showed little change (**Figure 3B**). There were no significant difference in the growth curves of the above four strains (**Figure 3C**). Next, the SM10λπ-PAO1 model was used to investigate the roles of *sdiA* ^+^, △*sdiA* and △*sdiA*/GadY^+^ in regulating conjugation. Overexpression of *sdiA* significantly decreased SM10λπ-PAO1 conjugation, while an obvious increase was observed in the *sdiA*-deficient group (**Figure 3D**). The inhibitory role of SdiA in bacterial conjugation is consistent with our previous study (Lu et al., 2017). Further analysis of the result of △*sdiA*/GadY^+^ group, it showed no difference with the △*sdiA* group (**Figure 3D**). For SM10λπ-EC600 conjugation, we subsequently added exogenous AHLs in this model and measured GadY’s role. As shown in **Figure 3E**, exogenous addition of 3-oxo-C12-HSL and C4-HSL rescued the positive regulation of GadY in conjugation. Taken together, the data from **Figures 2**, **3** suggest that GadY enhances bacterial conjugation by targeting SdiA and then attenuating the AHLs-SdiA signalling.

### Involvement of GadY and SdiA in regulating the global RP4 repressors KorA and KorB

**Figure 4:**
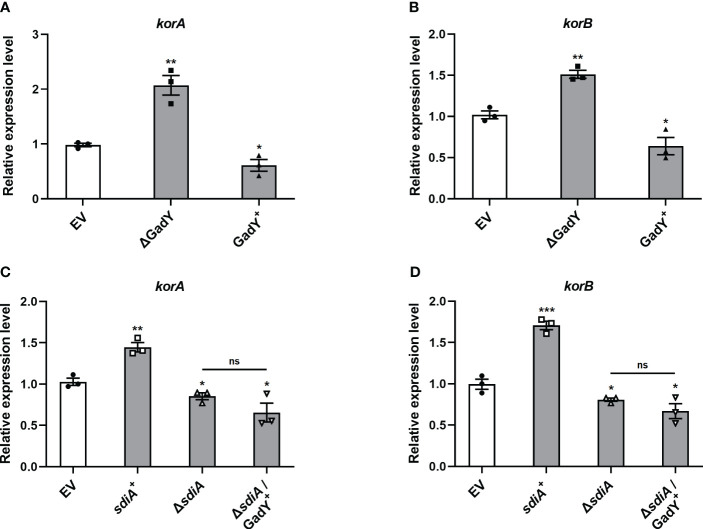
The mRNA expressions of global RP4 repressors KorA and KorB were regulated by GadY and SdiA. **(A, B)** *E*. *coli* SM10λπ EV, GadY^+^, and △GadY strains were grown in LB for 6 h respectively, then *korA* **(A)** and *korB* **(B)** expressions were examined by qRT-PCR. **(C, D)** *E*. *coli* SM10λπ EV, *sdiA* ^+^, △*sdiA*, and △*sdiA*/GadY^+^ strains were grown in LB for 6 h respectively, then *korA* **(C)** and *korB* **(D)** expressions were detected by qRT-PCR. Data are shown as mean ± SEM of at least three independent experiments. *, *P* < 0.05; **, *P* < 0.01; ***, *P* < 0.001; ns, non-significant.

The conjugation process is initiated by the assembly of a relaxosome at the transfer origin (*oriT*), which requires the interaction of transfer-related proteins such as TraJ and TraI (Pansegrau et al., 1990, 1993; Koraimann et al., 1996). Thus, we first examined the role of GadY in regulating TraJ and TraI mRNA expressions in the donor strain SM10λπ. The expression levels of traJ (**Supplementary Figure S1A**) and traI (**Supplementary Figure S1B**) were decreased in the GadY deletion mutant. However, both traJ and traI expressions were not significantly affected in the GadY overexpressing strain (**Supplementary Figures S1A, B**). Encoded in the IncP plasmid’s central control region, the KorA and KorB proteins are global repressors leading to a potent decrease in conjugative transfer (Balzer et al., 1992; Jagura-Burdzy and Thomas, 1994; Kostelidou et al., 1999). We subsequently measured KorA and KorB mRNA expressions in the GadY-deficient or overexpressing donor strains. As shown in **Figures 4A, B**, the results showed an obvious up-regulation of *korA* and *korB* expressions in the GadY deletion mutant, and a decrease in the GadY overexpressing strain. It suggests that GadY promotes bacterial conjugation by down-regulation of KorA and KorB. In **Figures 2** and **3**, we have demonstrated that GadY enhances conjugation by targeting SdiA. To further clarify whether SdiA involves in the effects of GadY on KorA and KorB, *sdiA* gene-deficient and overexpressing strains were used. As shown in **Figures 4C, D**, both *korA* and *korB* expressions were significantly up-regulated in *sdiA* ^+^ strain, but decreased in the △*sdiA* strain and △*sdiA*/GadY^+^ strain. The expressions of *korA* and *korB* in △*sdiA*/GadY^+^ strain had no difference with the △*sdiA* group (**Figures 4C, D**). These results indicate that the negative regulation of KorA and KorB by GadY depends on base-pairing with its target mRNA SdiA.

**Figure 5:**
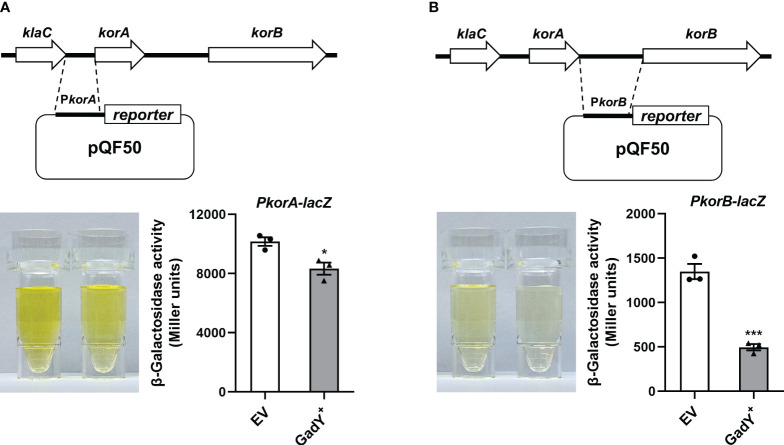
The promoter activities of *korA* and *korB* were affected by GadY. **(A, B)** Diagram of the *korA*-*korB* operon and the investigation of the promoter activities of *korA* **(A)** and *korB* **(B)**. The *E*. *coli* DH5α strains carrying the pQF50-*PkorA* **(A)** or pQF50-*PkorB* **(B)** reporter plasmid combined with pSTV28 (EV) or pSTV28-GadY^+^ (GadY^+^) were cultured at 37 °C for 6 h to the mid-log phase, and the β-galactosidase activity was measured. Data are shown as mean ± SEM of at least three independent experiments. *, *P* < 0.05; ***, *P* < 0.001.

The qRT-PCR results in **Figure 4** preliminarily showed GadY down-regulated *korA* and *korB* expressions at the transcriptional level. To further confirm this, we constructed a transcriptional *lacZ* fusion to the *korA* or *korB* promoter. The DNA sequence of *korA* or *korB* promoter, namely *PkorA* or *PkorB*, was inserted respectively upstream of the β-galactosidase gene *lacZ* in the pQF50 reporter plasmid (**Figures 5A, B**). Compared with the EV group, the β-galactosidase activity from the *PkorA*-*lacZ* fusion was inhibited by GadY (**Figure 5A**). More significantly, GadY greatly impaired the activity from the *PkorB*-*lacZ* fusion (**Figure 5B**). These findings establish that the transcriptional activities of *korA* and *korB* promoter regions are repressed by GadY.

### GadY promotes quinolone antibiotics-induced conjugation

**Figure 6:**
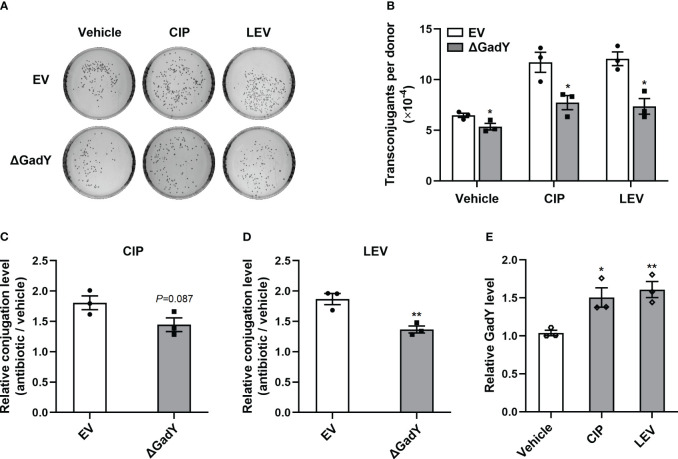
Quinolone antibiotics induced up-regulated expression of GadY to promote SM10λπ-PAO1 conjugation. **(A, B)** *E. coli* SM10λπ (EV or △GadY) were treated with sub-MIC of CIP, LEV or untreated (vehicle) for 6 h at 37°C, then were mated with PAO1 (1×10^7^ CFU/ml each) for 6 h, and transconjugant colonies numbers were counted. **(C, D)** The relative conjugation levels by calculating the transconjugant ratio of antibiotic to vehicle were analyzed, and CIP **(C)**, LEV **(D)**. **(E)** SM10λπ was treated with sub-MIC of CIP, LEV or untreated (vehicle) for 6 h at 37°C, and the expression levels of GadY was examined by qRT-PCR. Data are shown as mean ± SEM of at least three independent experiments. *, *P* < 0.05; **, *P* < 0.01.

To further investigate the roles of GadY in antibiotics-induced conjugation, several types of antibiotics including ciprofloxacin (CIP), levofloxacin (LEV), gentamicin (GM), azithromycin (AZM) and chloramphenicol (CM) were separately added to the SM10λπ-PAO1 conjugation model. These antibiotics positively regulate bacterial conjugation, as demonstrated in our previous studies (Shun-Mei et al., 2018; Lu et al., 2017a). In the EV group, the conjugations under the above antibiotics treatments were enhanced, while deletion of GadY significantly decreased the numbers of transconjugants under CIP, LEV or GM treatment (**Figures 6A, B**; **Supplementary Figure S2A**). In order to demonstrate the roles of GadY in SM10λπ-PAO1 conjugation under antibiotics treatments more clearly, relative conjugation levels by calculating the transconjugant ratio of antibiotic to vehicle were analyzed. As shown in **Figures 6C, D**, the increase of LEV on SM10λπ-PAO1 conjugation was much inhibited in △GadY group than in EV group, and there was also a slight decline under CIP treatment. However, the relative conjugation levels were not affected by GadY deletion under GM, AZM or CM treatment (**Supplementary Figures S2B-D**). The role of antibiotics in the expression of GadY in SM10λπ was finally examined, and we found the presence of either CIP or LEV significantly increased GadY expression (**Figure 6E**), but no changes were observed in GM, AZM or CM group (**Supplementary Figure S2E**). These results indicate that quinolone antibiotics mediate the up-regulated expression of GadY, which positively regulates SM10λπ-PAO1 conjugation.

## Discussion

**Figure 7:**
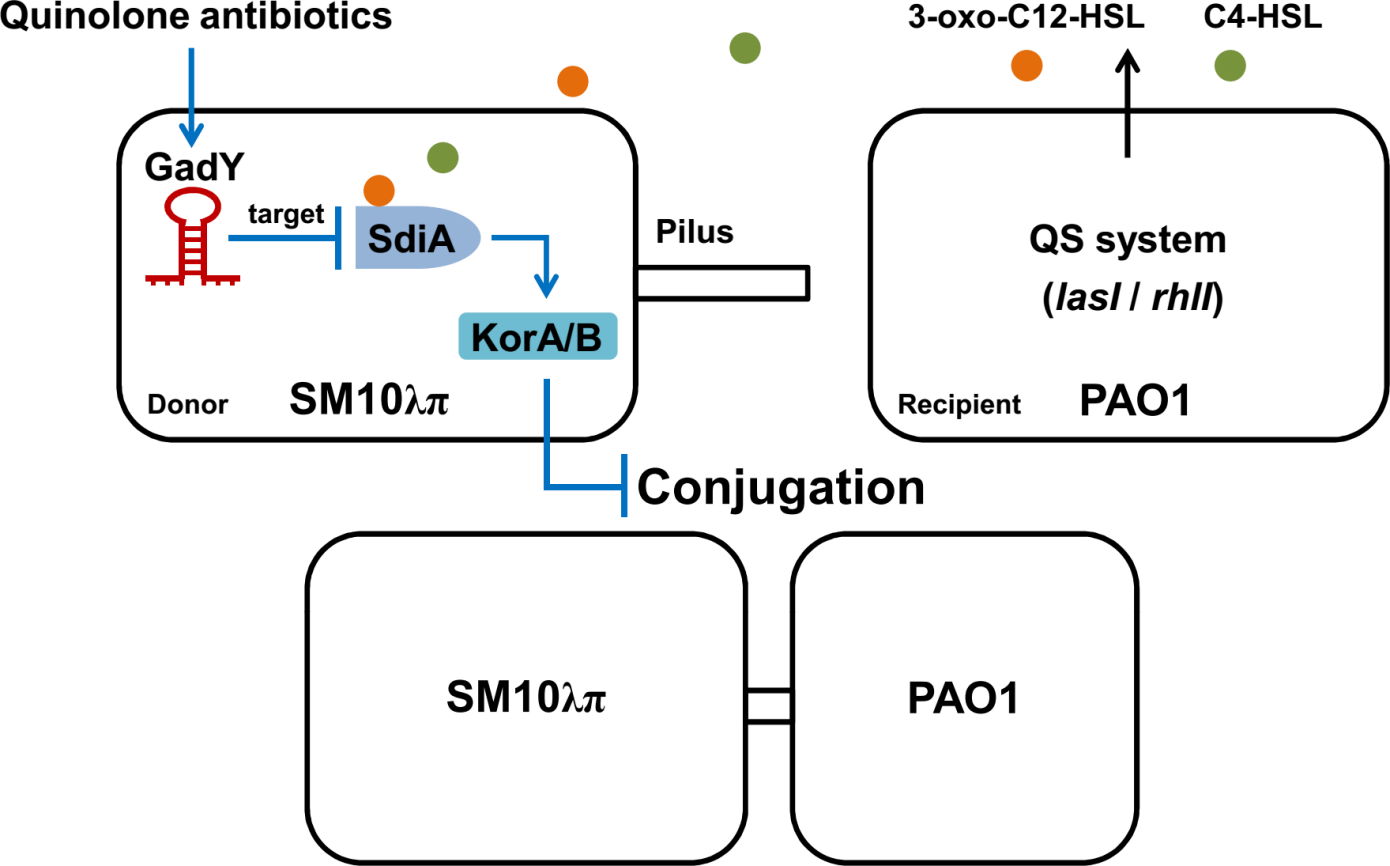
A working model of how GadY promotes SM10λπ-PAO1 conjugation by targeting SdiA under quinolone antibiotics treatments. GadY expression in donor strain SM10λπ was up-regulated by ciprofloxacin or levofloxacin treatment. Then GadY promoted SM10λπ-PAO1 conjugation by base-pairing with its target mRNA SdiA, an orphan LuxR-type receptor that responded to exogenous AHLs produced by the recipient strain PAO1. The transcription of the global RP4 repressors KorA and KorB was subsequently inhibited by GadY.

In this study, the Hfq-binding sRNA GadY in donor strain *E. coli* SM10λπ is found to be involved in regulating bacterial conjugation (**Figure 7**). GadY promoted SM10λπ-PAO1 conjugation by base-pairing with its target mRNA SdiA. In *E. coli*, the orphan LuxR-type receptor SdiA responds to exogenous AHLs from other bacterial species like *P. aeruginosa* (Yamamoto et al., 2001). Given this, we further clarified that the effects of GadY on SM10λπ-PAO1 conjugation depended on AHLs-SdiA signalling. Additional studies found GadY decreased the expression levels of KorA and KorB mRNA by targeting SdiA. The expression of GadY in donor strain was up-regulated by CIP or LEV treatment, and it promoted quinolone-induced conjugation. Therefore, the Hfq-binding sRNA GadY from donor strain *E. coli* is a novel regulator involved in bacterial conjugation.

GadY is an Hfq-binding sRNA and has been reported in the past as a potent regulator of acid response in *E. coli* (Opdyke et al., 2004). Other biological functions of GadY are currently still unknown. Given that its chaperone Hfq plays important roles in bacterial conjugation by regulating the transcript stability of transfer-related genes *traM* and *traJ* (Will and Frost, 2006), GadY’s roles in bacterial conjugation arouse our interest. Based on the SM10λπ-PAO1/EC600 conjugation models established in our previous studies (Lu et al., 2017b; Xiong et al., 2021), gadY gene-deficient and overexpressing donor strains (SM10λπ) were thus constructed, and PAO1 or EC600 were used as recipient strains. Intriguingly, GadY positively enhanced the conjugation between SM10λπ and PAO1, but had no significance in SM10λπ-EC600 conjugation (**Figure 1**). We speculate that the different roles of GadY in regulating SM10λπ’s conjugation with PAO1 or EC600 may be related to the AHLs produced by recipient strains. PAO1 has autoinducer synthase from QS system to synthesize various AHLs including 3-oxo-C12-HSL, C4-HSL, quinolones and IQS signals (Papenfort and Bassler, 2016; García-Reyes et al., 2020). In contrast to PAO1, EC600 could not produce AHLs because it lacks AHLs synthase.

A previous study in our group has demonstrated that 3-oxo-C12-HSL and C4-HSL from PAO1 activate the SdiA receptor of SM10λπ, which results in the attenuation of SM10λπ-PAO1 conjugation (Lu et al., 2017b). SdiA is an orphan receptor in bacterial species such as Escherichia, Klebsiella, Salmonella, and Shigella, which responds to a wide range of exogenous AHLs (Smith et al., 2011). These organisms are nonAHL-producing bacteria because they lack AHLs synthase (Mayer et al., 2023). Therefore, the different roles of GadY in SM10λπ-PAO1/EC600 conjugation may be closely related to the AHLs-SdiA signalling. Base-pairing with its target mRNAs is an important way for sRNA to regulate various genes (Desgranges et al., 2019; Jørgensen et al., 2020). Using a GFP reporter system including WT and Mut experiments, SdiA was surprisingly demonstrated as a direct target of GadY (**Figure 2**). Adding AHL in the SM10λπ-EC600 model rescued GadY’s roles in conjugation (**Figure 3**). It indicates that GadY-mediated regulation of bacterial conjugation depends on the AHLs-SdiA signalling between the donor and recipient cells.

The interaction between sRNA and its target mRNA can affect translation initiation, transcription termination or mRNA stability. The binding sites of sRNA can occur at different regions of the target mRNAs, such as the 5’ untranslated region (5’-UTR), 3’ untranslated region (3’-UTR), and the coding region (Waters and Storz, 2009). Most of the intergenic sRNAs frequently bind to the 5’-UTR of the target mRNAs where the ribosome accommodation usually appears (Storz et al., 2011). The coding sequence is also a usual region for some sRNA to interact with its target mRNA. The Hfq-binding sRNA DsrA in *E. coli* base-pairs far downstream in the coding sequence of rbsD and induces mRNA decay (Lalaouna et al., 2015). MicC as an Hfq-associated sRNA in Salmonella binds to the coding sequence of ompD to accelerate RNase E-dependent mRNA decay (Pfeiffer et al., 2009). The base-pairing region of GadY was around 552~582 nt from the start codon of sdiA (**Figure 2**). Given the coding sequence of sdiA is 723 nt, GadY interacted downstream of the coding region. Likewise, GadY is also an Hfq-binding sRNA as DsrA and MicC, and the sites for GadY interacting with SdiA are comprised of a poly-U tail, an AU-rich single-stranded region which is often required for Hfq binding on sRNA (Link et al., 2009). It suggests that base-paring with the coding sequence and then affecting the stability and translation of mRNA may be an alternative way for Hfq-binding sRNA to regulate its target.

The donor strain *E. coli* SM10λπ used in our conjugation model carries a chromosomally integrated IncP-1α plasmid RP4. The transfer of conjugative DNA, a pUCP24T plasmid, mediated by RP4 starts at oriT. In this process, an oriT-recognizing protein TraJ is encoded by the traJ gene and is essential for the initiation of transfer DNA replication. The TraJ protein binds oriT and begins the formation of a relaxosome, and then the TraI protein, a key component of the relaxosome, combines the TraJ oriT complex to promote the transfer process (Fürste et al., 1989; Pansegrau et al., 1994). However, overexpression of GadY did not significantly affect the expressions of traJ and traI, although GadY deletion had some changes (**Supplementary Figure S1**). It suggests GadY’s roles in conjugation are not achieved by regulating TraJ and TraI.

In addition to transfer-related proteins, the global transcriptional repressors KorA and KorB are major factors in controlling the regulatory circuit of RP4 plasmid (Young et al., 1987). KorA has a dimerization module shared by the RP4 protein TrbA, another global repressor which inhibits the transcription of *trfA* (Jagura-Burdzy et al., 1992; Jagura-Burdzy and Thomas, 1994). KorA also causes derepression of the promoter for *trbA* (Jagura-Burdzy and Thomas, 1994). In conjunction with KorA, KorB works as a regulator protein in modulating transfer genes expression by suppressing the transcription of the *trfA*, *kilA* and *korAB* operons (Schmidhauser et al., 1989; Bingle et al., 2005). We found that the *korA* and *korB* mRNA expressions were affected by GadY and SdiA (**Figure 4**). The transcriptional activities of *korA* and *korB* promoter regions were also analyzed. It was reported that *korA* and *korB* are co-transcribed from the upstream promoter of *korA*, and *korB* is primarily expressed in an operon with *korA* (Bechhofer and Figurski, 1983; Smith and Thomas, 1984; Bechhofer et al., 1986). However, when a heterologous promoter upstream of *korB* is present, *PkorA* is nonessential to *korB* transcription (Bechhofer et al., 1986; Young et al., 1987). Our results from the β-galactosidase assays showed that *PkorB* has weaker promoter activity than *PkorA*, and GadY inhibited the activities of both of them (**Figure 5**). The results from **Figures 4**, **5** suggest that the inhibitory effects on KorA and KorB enable GadY to promote bacterial conjugation, and this process depends on the base-pairing between GadY and its target mRNA SdiA.

As a common mechanism of horizontal transfer of ARGs, plasmid-mediated conjugation contributes to the rapid spread of antibiotic resistance among bacteria. Therefore, we finally investigated the roles of GadY in conjugation induced by different antibiotics, including CIP, LEV, GM, AZM and CM. A previous work in our group found sub-minimal inhibitory concentrations (sub-MIC) of CIP or LEV treating the donor strain SM10λπ significantly increased conjugation frequency (Shun-Mei et al., 2018). We also found PAO1 treated with GM, AZM or CM promoted SM10λπ-PAO1 conjugation (Lu et al., 2017a). Thus, these five antibiotics were used in the current study. The results showed that GadY deletion decreased SM10λπ-PAO1 conjugation under sub-MIC CIP or LEV treatment, but had little influence on GM, AZM or CM group (**Figure 6**; **Supplementary Figure S2**). It may be related to the different ways in which the several types of antibiotics regulate bacterial conjugation.

CIP and LEV are synthetic quinolone antibiotics with a broad spectrum of antimicrobial activity for treating various types of infections in humans (Bolon, 2009). However, the increasing emergence of antibiotic resistance compromised their use. The acquisition of quinolone resistance has multiple causes. Plasmid-mediated conjugation is one of the main resistance mechanisms, besides genomic mutations of the bacteria (Strahilevitz et al., 2009). Our previous work showed treating the donor stains SM10λπ with sub-MIC (1/8-1/32 MIC) of CIP or LEV promote plasmid transfer into the recipient strains PAO1 through conjugation (Shun-Mei et al., 2018). An earlier study also found low concentrations of CIP increase the transfer frequencies of resistance plasmids (Ohlsen et al., 2003). GM, AZM and CM have been identified as QS-inhibiting antibiotics which reduce *P. aeruginosa* AHLs production (Skindersoe et al., 2008; Lu et al., 2017a; Zeng et al., 2016). This suggests GM, AZM and CM positively regulate SM10λπ-PAO1 conjugation by suppressing the AHLs-SdiA signalling between the donor and recipient cells. Thus, CIP and LEV promote bacterial conjugation by influencing the donor strain SM10λπ, while GM, AZM and CM function by affecting the recipient strain PAO1. Intriguingly, our further study found sub-MIC CIP or LEV treatment significantly increased GadY expression in donor strains, while GM, AZM or CM treatment did not (**Figure 6**; **Supplementary Figure S2**). It suggests that the up-regulated expression of GadY in donor strains by quinolone antibiotics increases SM10λπ-PAO1 conjugation.

In conclusion, a novel role for the Hfq-binding sRNA GadY in regulating conjugation between *E. coli* and other bacteria is demonstrated in this study. Positively induced by quinolone antibiotics, GadY involves in regulating SM10λπ-PAO1 conjugation by targeting the orphan LuxR-type receptor SdiA, and then decreases the expressions of global repressors KorA and KorB. Thus, our findings disclose a novel function of the Hfq-binding sRNA GadY and shed new light on developing a promising approach to counter antibiotic resistance by inhibition of conjugation.

## Data availability statement

The original contributions presented in the study are included in the article/**Supplementary Material**. Further inquiries can be directed to the corresponding authors.

## Author contributions

SZ: Data curation, Formal Analysis, Methodology, Project administration, Writing – original draft. JL: Data curation, Formal Analysis, Project administration, Writing – original draft. QL: Data curation, Formal Analysis, Project administration, Writing – original draft. ML: Data curation, Formal Analysis, Writing – review & editing. RY: Data curation, Formal Analysis, Writing – review & editing. YL: Resources, Writing – review & editing. XM: Formal Analysis, Writing – review & editing. YC: Formal Analysis, Writing – review & editing. CS: Resources, Writing – review & editing. JZ: Resources, Writing – review & editing. BH: Conceptualization, Supervision, Writing – review & editing. CC: Conceptualization, Supervision, Writing – review & editing. JP: Conceptualization, Funding acquisition, Project administration, Supervision, Writing – original draft, Writing – review & editing.
