# Supplementary material for: Small RNA GadY in Escherichia coli enhances conjugation system of IncP-1 by targeting SdiA
Source: Front Cell Infect Microbiol. 2024 Jul 23;14:1445850. doi: 10.3389/fcimb.2024.1445850 (PMC11300174; doi:10.3389/fcimb.2024.1445850)
Supplement: Supplementary file 1 [file DataSheet_1.docx]

**Small RNA GadY in *Escherichia coli* enhances conjugation system of IncP-1 by targeting SdiA**

Shebin Zhang ^1,2#^, Jiao Long ^1,2#^, Qiwei Li ^1,2#^, Mo Li^1,2^, Ruiqi Yu^1,2^, Yang Lu^1,2^, Xingyan Ma^4^, Yimei Cai^1,2^, Cong Shen^1,2,3^, Jianming Zeng^1,2,3^, Bin Huang^4*^, Cha Chen^1,2*^, Jieying Pu^1,2,3*^

^1^ The Second Clinical Medical College, Guangzhou University of Chinese Medicine, State Key Laboratory of Traditional Chinese Medicine Syndrome, Guangdong Provincial Hospital of Chinese Medicine, Guangzhou, China

^2^ Department of Laboratory Medicine, The Second Affiliated Hospital of Guangzhou University of Chinese Medicine, Guangzhou, China

^3^ Guangdong Provincial Key Laboratory of Research on Emergency in TCM, Guangzhou, China

^4^ Department of Laboratory Medicine, The First Affiliated Hospital of Sun Yat-sen University, Guangzhou, China

^*^Correspondence to:

Bin Huang, Department of Laboratory Medicine, The First Affiliated Hospital of Sun Yat-sen University, Guangzhou, China. E-mail: huangb3@mail.sysu.edu.cn

Cha Chen, The Second Clinical Medical College, Guangzhou University of Chinese Medicine, State Key Laboratory of Traditional Chinese Medicine Syndrome, Guangdong Provincial Hospital of Chinese Medicine, Guangzhou, China. E-mail: chencha906@163.com

Jieying Pu, The Second Clinical Medical College, Guangzhou University of Chinese Medicine, State Key Laboratory of Traditional Chinese Medicine Syndrome, Guangdong Provincial Hospital of Chinese Medicine, Guangzhou, China. E-mail: pujy1105@163.com

^#^The first three authors contributed equally to this work.

**Table S1** Bacterial strains and plasmids

| **Strains / Plasmids** | **Characteristics** | **Source** |
| --- | --- | --- |
| ***Escherichia coli*** |  |  |
| SM10λπ | Wild-type strain, GM^S^, AMP^S^ | Our lab |
| SM10λπ △GadY | Deficiency of *gadY*, GM^S^, AMP^S^ | This work |
| SM10λπ △*sdiA* | Deficiency of *sdiA*, GM^S^, AMP^S^ | Our lab^(1)^ |
| DH5α | Wild-type strain, GM^S^, AMP^S^ | Our lab |
| EC600 | Wild-type strain, GM^S^, RIF^R^ | Our lab |
| ***Pseudomonas*** |  |  |
| PAO1 | Wild-type strain, GM^S^, AMP^R^ | Our lab |
| ***Plasmids*** |  |  |
| pSTV28 (EV) | Control plasmid, containing P*_lac_* promoter, CM^R^ | Our lab |
| pSTV28-GadY^+^ | SM10λπ *gadY* overexpressing plasmid, controlled by P*_lac_* promoter, CM^R^ | This work |
| pSTV28-mut-GadY^+^ | SM10λπ mutant *gadY* overexpressing plasmid, controlled by P*_lac_* promoter, CM^R^ | This work |
| pSTV28-*sdiA*^+^ | SM10λπ *sdiA* overexpressing plasmid, controlled by P*_lac_* promoter, CM^R^ | Our lab^(1)^ |
| pKD3 | oriR6K, FRT::cat::FRT template plasmid, CM^R^ | Our lab |
| pKD46 | oriR101 repA101ts P-araB-gam-bet-exo, AMP^R^ | Our lab |
| pCP20 | pSC101 temperature-sensitive repliconts, Flp (λ Rp), cI857, CM^R^, AMP^R^ | Our lab |
| pUCP24T | Shuttle vector, 370 bp oriT fragment from pCVD442 cloned into pUCP24, ori1600, GM^R^ | Our lab |
| pUCP30T-*gfp* | Control plasmid, *gfp* controlled by P*_lac_* promoter, GM^R^ | Our lab^(2,3)^ |
| pUCP30T-*sdiA-gfp* | Containing transcription fusion of *sdiA-gfp*, which controlled by P*_lac_* promoter, GM^R^ | This work |
| pUCP30T-mut-*sdiA-gfp* | Containing transcription fusion of mut*-sdiA-gfp*, which controlled by P*_lac_* promoter, GM^R^ | This work |
| pQF50 | Reporter plasmid containing the *lacZ* gene, AMP^R^ | Our lab^(2)^ |
| pQF50-*PkorA* | pQF50 derivative reporter plasmid, controlled by the constitutive *korA* promoter, AMP^R^ | This work |
| pQF50-*PkorB* | pQF50 derivative reporter plasmid, controlled by the constitutive *korB* promoter, AMP^R^ | This work |
|  |  |  |

GM, AMP, CM and RIF stand for gentamicin, ampicillin, chloramphenicol and rifampin, respectively. S, sensitive; R, resistant.

**References:**

1. Lu Y, Zeng J, Wu B, E S, Wang L, Cai R, Zhang N, Li Y, Huang X, Huang B, Chen C. Quorum sensing N-acyl homoserine lactones-SdiA suppresses *Escherichia coli*-*Pseudomonas aeruginosa* conjugation through inhibiting *traI* expression. Front Cell Infect Microbiol, 2017, 7:7.
2. Lu Y, Li H, Pu J, Xiao Q, Zhao C, Cai Y, Liu Y, Wang L, Li Y, Huang B, Zeng J, Chen C. Identification of a novel RhlI/R-PrrH-LasI/Phzc/PhzD signalling cascade and its implication in *P. aeruginosa* virulence. Emerg Microbes Infect, 2019, 8(1):1658-1667.
3. Pu J, Zhang S, He X, Zeng J, Shen C, Luo Y, Li H, Long Y, Liu J, Xiao Q, Lu Y, Huang B, Chen C. The small RNA AmiL regulates quorum sensing-mediated virulence in *Pseudomonas aeruginosa* PAO1. Microbiol Spectr, 2022, 10(2):e0221121.

**Table S2** Sequences of cloning primers and qRT-PCR primers

| **Name** | **Purpose** | **Sequence (5’→3’)** | **Source** |
| --- | --- | --- | --- |
| ***Cloning primers*** | | |  |
| GadY-M1 | △GadY | CTGCCAGATTTGGTAGGACC | This work |
| GadY-M2 | △GadY | AGAGTATCAGGAGCGATCG | This work |
| GadY-knock-F | △GadY | TTTATAAAAAAATGGCTGATCTTATTTCCAGTAAAAGTTATATTTAACTTTGTAGGCTGGAGCTGCTTCG | This work |
| GadY-knock-R | △GadY | ACTGACCGTTCTGCGGAAGGAATAAGATTATAGAGTTTTACTCAGACATAATGGGAATTAGCCATGGTCC | This work |
| In28-*gadY*-F | GadY^+^ | TATGACCATGATTACGAATTACTGAGAGCACAAAGTTTCC | This work |
| In28-*gadY*-R | GadY^+^ | ACGACGGCCAGTGCCAAGCTAAAAAAACCCGGCATAGGGG | This work |
| In30-*sdiA*-F | *sdiA-gfp* | GCTCTAGAATGCGCGAAAGTCTGATGGCTCT | This work |
| In30-*sdiA*-R | *sdiA-gfp* | CATGCCATGGAATCATCGCTATCTCTGCTG | This work |
| mut-*gadY*-P1 | Mut GadY^+^ | AGGTGGACGGCGGAAGGGAAGCTTGGCACTGGCCGT | This work |
| mut-*gadY*-P4 | Mut GadY^+^ | TCCCTTCCGCCGTCCACCTCAGAATTTCTTTGATAGTCTGCCGTCTCCA | This work |
| mut-*sdiA*-P1 | mut*- sdiA-gfp* | CCTATGCCGGGTTTTTTTAAACATCAGCAGAGATAG | This work |
| mut-*sdiA*-P4 | mut*- sdiA-gfp* | AAAAAAACCCGGCATAGGGGACCGGGAAGAGTTCGCGCTTGCTGAAAT | This work |
| mut-P2 | fusion | GGAGAGGCGGTTTGCGTA | This work |
| mut-P3 | fusion | TACGCAAACCGCCTCTCC | This work |
| In50-*korA*-F | *PkorA* | CCGGGGATCTCTAGAAGCTTAAGCGGCAAGAGACGAAAGC | This work |
| In50-*korA*-R | *PkorA* | TACCCTCTAGCTAGAAGCTTGGATAACACCCATAATTTGC | This work |
| In50-*korB*-F | *PkorB* | CCGGGGATCTCTAGAAGCTTAGCTTGAGGCGTACAGCA | This work |
| In50-*korB*-R | *PkorB* | TACCCTCTAGCTAGAAGCTTTGGGAAATCTCCATCTTCGT | This work |
| ***qRT-PCR primers*** | | |  |
| *rpoD*-F | qRT-PCR | GGGATCAACCAGGTTCAATG | Our lab^(1)^ |
| *rpoD*-R | qRT-PCR | GGTGCCAGATCTTCTTCTGC | Our lab^(1)^ |
| *gadY*-F | qRT-PCR | CAAAGTTTCCCGTGCCAA | Our lab^(1)^ |
| *gadY*-R | qRT-PCR | CATAGGGGACCGGGAAGA | Our lab^(1)^ |
| *sdiA*-F | qRT-PCR | ATTCAGCGAAGCACAGCC | This work |
| *sdiA*-R | qRT-PCR | AACGCATCAGAGCCATCA | This work |
| *korA*-F | qRT-PCR | GCTTACCGAAAGCCAGTTCCAG | Our lab^(2)^ |
| *korA*-R | qRT-PCR | GCAAGTTCTTGTCCTCGAACGC | Our lab^(2)^ |
| *korB*-F | qRT-PCR | AAGGAAAAGGGCGCGAAGGAG | Our lab^(2)^ |
| *korB*-R | qRT-PCR | TCGATGAGCGCGACCAGTTTC | Our lab^(2)^ |
| *traJ*-F | qRT-PCR | CGAACGAAGAGCGATTGAGG | Our lab^(2)^ |
| *traJ*-R | qRT-PCR | TCGTCGGTGAGCCAGAGTTT | Our lab^(2)^ |
| *traI*-F | qRT-PCR | ATCACGAAGGGAACCATCATC | Our lab^(2)^ |
| *traI*-R | qRT-PCR | TTGAACTCTGCTGTGCCGTTGAC | Our lab^(2)^ |

**References:**

1. Ma X, Zhang S, Xu Z, Li H, Xiao Q, Qiu F, Zhang W, Long Y, Zheng D, Huang B, Chen C, Lu Y. SdiA improves the acid tolerance of *E. coli* by regulating gadW and gadY expression. Front Microbiol, 2020, 11:1078.
2. Xiong R, Liu Y, Pu J, Liu J, Zheng D, Zeng J, Chen C, Lu Y, Huang B. Indole inhibits IncP-1 conjugation system mainly through promoting korA and korB expression. Front Microbiol, 2021, 12:628133.

**FIGURE S1**

**
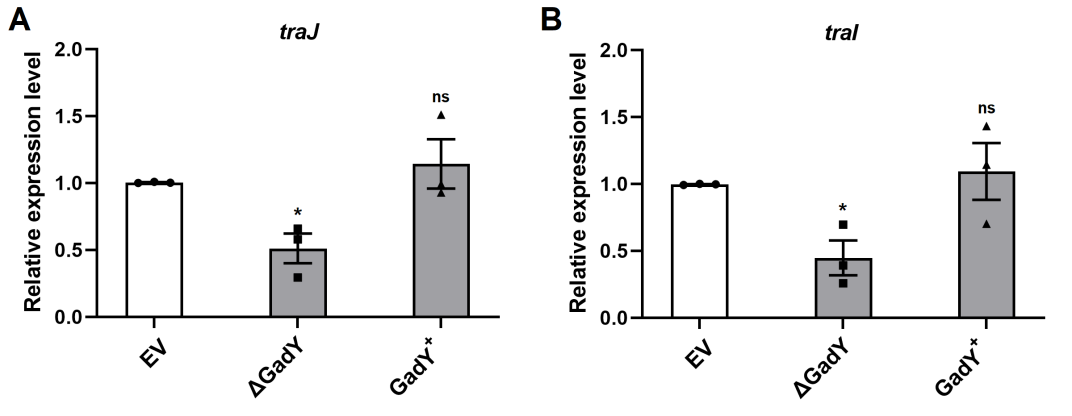
**

GadY had little effect on *traJ* and *traI* expressions. **(A-B)** *E. coli* SM10λπ EV GadY^+^, and △GadY strains were grown in LB for 6 h respectively, then *traJ* (A) and *traI* (B) expressions were detected by qRT-PCR. Data are shown as mean ± SEM of at least three independent experiments. *, *P* < 0.05; ns, non-significant.

**FIGURE S2**
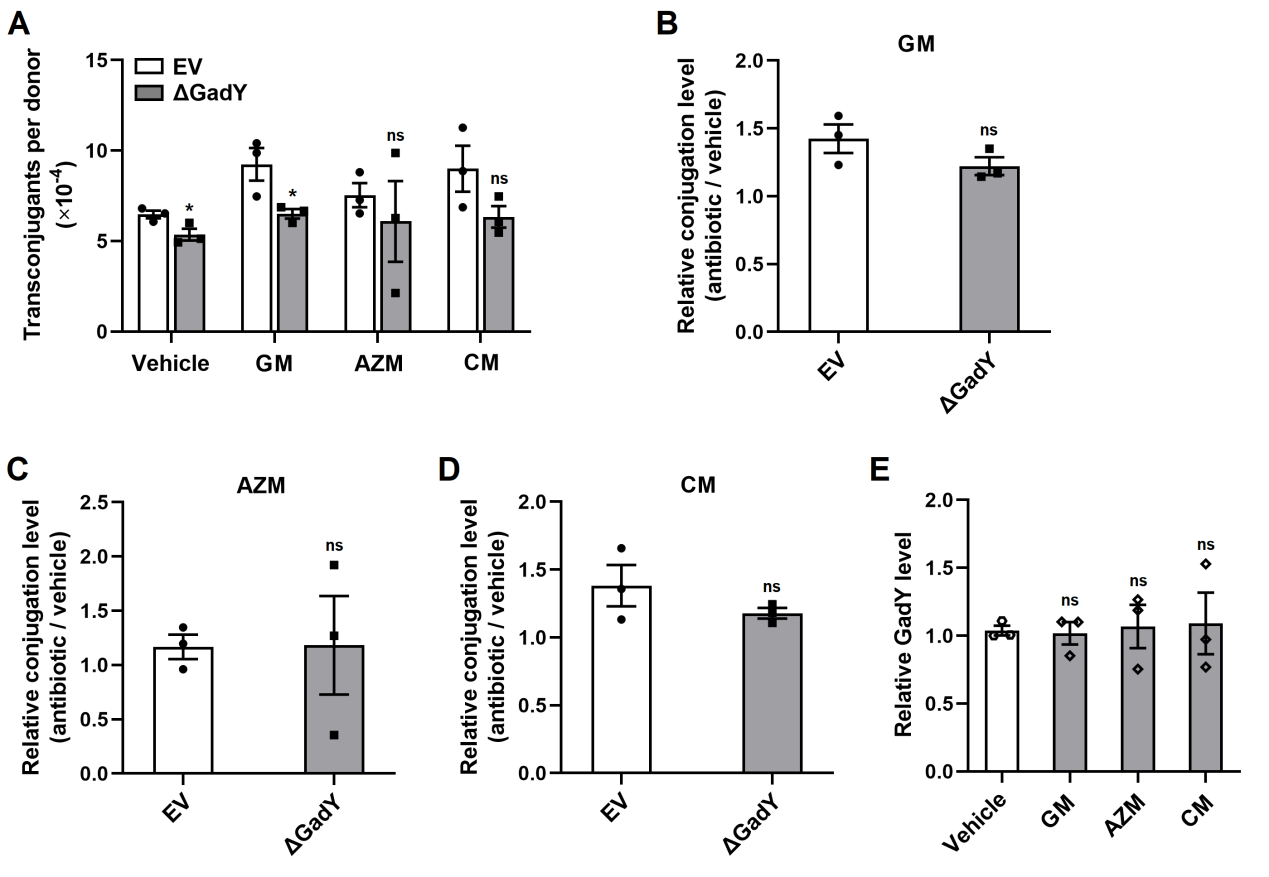
GadY had little effect on gentamicin-, azithromycin-, or chloramphenicol-induced SM10λπ-PAO1 conjugation. **(A)** *E. coli* SM10λπ EV or △GadY strains were treated with sub-MIC of GM, AZM, CM or untreated (vehicle) for 6 h at 37℃, then were mated with PAO1 (1×10^7^ CFU/ml each) for 6 h, and transconjugant colonies numbers were counted. **(B-D)** The relative conjugation levels by calculating the transconjugant ratio of antibiotic to vehicle were analyzed, and GM (B), AZM (C), CM (D). **(E)** SM10λπ was treated with sub-MIC of GM, AZM, CM or untreated (vehicle) for 6 h at 37℃, and GadY expression was examined by qRT-PCR. Data are shown as mean ± SEM of at least three independent experiments. *, *P* < 0.05; ns, non-significant.
